# Autophagy-Regulating microRNAs and Cancer

**DOI:** 10.3389/fonc.2017.00065

**Published:** 2017-04-18

**Authors:** Devrim Gozuacik, Yunus Akkoc, Deniz Gulfem Ozturk, Muhammed Kocak

**Affiliations:** ^1^Molecular Biology, Genetics and Bioengineering Program, Faculty of Engineering and Natural Sciences, Sabanci University, Istanbul, Turkey; ^2^Center of Excellence for Functional Surfaces and Interfaces for Nano Diagnostics (EFSUN), Sabanci University, Istanbul, Turkey

**Keywords:** autophagy, microRNA, post-transcriptional control, cancer growth, metastasis, chemotherapy, radiotherapy, biomarker

## Abstract

Macroautophagy (autophagy herein) is a cellular stress response and a survival pathway that is responsible for the degradation of long-lived proteins, protein aggregates, as well as damaged organelles in order to maintain cellular homeostasis. Consequently, abnormalities of autophagy are associated with a number of diseases, including Alzheimers’s disease, Parkinson’s disease, and cancer. According to the current view, autophagy seems to serve as a tumor suppressor in the early phases of cancer formation, yet in later phases, autophagy may support and/or facilitate tumor growth, spread, and contribute to treatment resistance. Therefore, autophagy is considered as a stage-dependent dual player in cancer. microRNAs (miRNAs) are endogenous non-coding small RNAs that negatively regulate gene expression at a post-transcriptional level. miRNAs control several fundamental biological processes, and autophagy is no exception. Furthermore, accumulating data in the literature indicate that dysregulation of miRNA expression contribute to the mechanisms of cancer formation, invasion, metastasis, and affect responses to chemotherapy or radiotherapy. Therefore, considering the importance of autophagy for cancer biology, study of autophagy-regulating miRNA in cancer will allow a better understanding of malignancies and lead to the development of novel disease markers and therapeutic strategies. The potential to provide study of some of these cancer-related miRNAs were also implicated in autophagy regulation. In this review, we will focus on autophagy, miRNA, and cancer connection, and discuss its implications for cancer biology and cancer treatment.

## Introduction

MicroRNAs (miRNAs) are small RNAs that play a key role in the regulation of gene expression. miRNAs do not code for proteins, but they control stability and translation of messenger RNAs (mRNAs) of protein-coding genes, and change abundance of proteins that are encoded by them. By this way, miRNAs modulate and orchestrate cellular pathways, including cell growth, differentiation, apoptosis, and migration pathways (1–3). Around 2,000 unique miRNAs were discovered in man and their numbers are growing.

Dysregulation of miRNA expression often correlates with human diseases. Up- or downregulation of miRNAs was reported in several cancer types as well. miRNA abnormalities contribute to various stages of cancer formation and progression, and even determine resistance to cancer treatment. Differential expression of miRNAs between tumors and their corresponding normal tissues led them to be introduced as potent cancer markers. Changes in specific miRNA levels were reported in almost all types of malignancies, including lung cancer, colon cancer, pancreatic cancer, breast cancer, and leukemia (4–8).

Autophagy is a highly conserved cellular mechanism that allows digestion and recycling of long-lived proteins, protein aggregates, intracellular pathogens, and even whole organelles, such as mitochondria. Active at a basal level in all cell types, autophagy is rapidly upregulated under stress conditions. Being a key guardian of cellular homeostasis, abnormalities of autophagy almost invariably lead to health problems, including cancer (9–13). A growing number of studies that were published in the last couple of years underline the importance of miRNAs in autophagy regulation. In this review article, we will briefly summarize miRNA and autophagy pathways and analyze emerging connections and correlations between autophagy, miRNAs, and cancer.

### MicroRNAs

MicroRNAs constitute an evolutionary conserved family of single-stranded, non-coding RNA molecules. These small RNAs are 17–25 nt in length. They control biological events through post-transcriptional gene silencing (14). miRNAs are found in a wide range of living organisms, e.g., from plants to mammals, providing evidence that gene expression control by miRNAs is an ancient mechanism (15). Computational predictions revealed that more than 60% of all human genes contain potential miRNA-binding sites; hence, all these genes might be subject to regulation by these small RNAs (16).

In the genome, miRNA genes and gene clusters can be found in both intergenic and intronic regions (6, 17). miRNAs residing in the same cluster might share the same transcriptional regulatory units. Hence, miRNAs may be expressed as polycistronic transcripts, allowing a coordinated expression pattern for functionally related miRNAs (18). Cellular levels of intronic miRNAs usually depend on the expression of the host protein-coding gene. Isolated miRNA genes exist as well; these genes possess their own promoters and can be expressed independently (18).

Long primary miRNAs or pri-miRNAs are 60–70 nt length RNA transcripts that are generally transcribed from miRNA genes in an RNA polymerase II (pol II)-dependent manner (19). However, transcription of some miRNA types may depend on RNA polymerase III (pol III) (20). Like protein-coding mRNAs, primary miRNA transcripts also contain a 5′ cap and a 3′ poly-A tail. miRNA may also be subject to splicing. Pri-miRNAs transcribed by RNA pol II may sometimes generate more than one functional miRNAs from a single pri-miRNA transcript (19).

**Figure 1:**
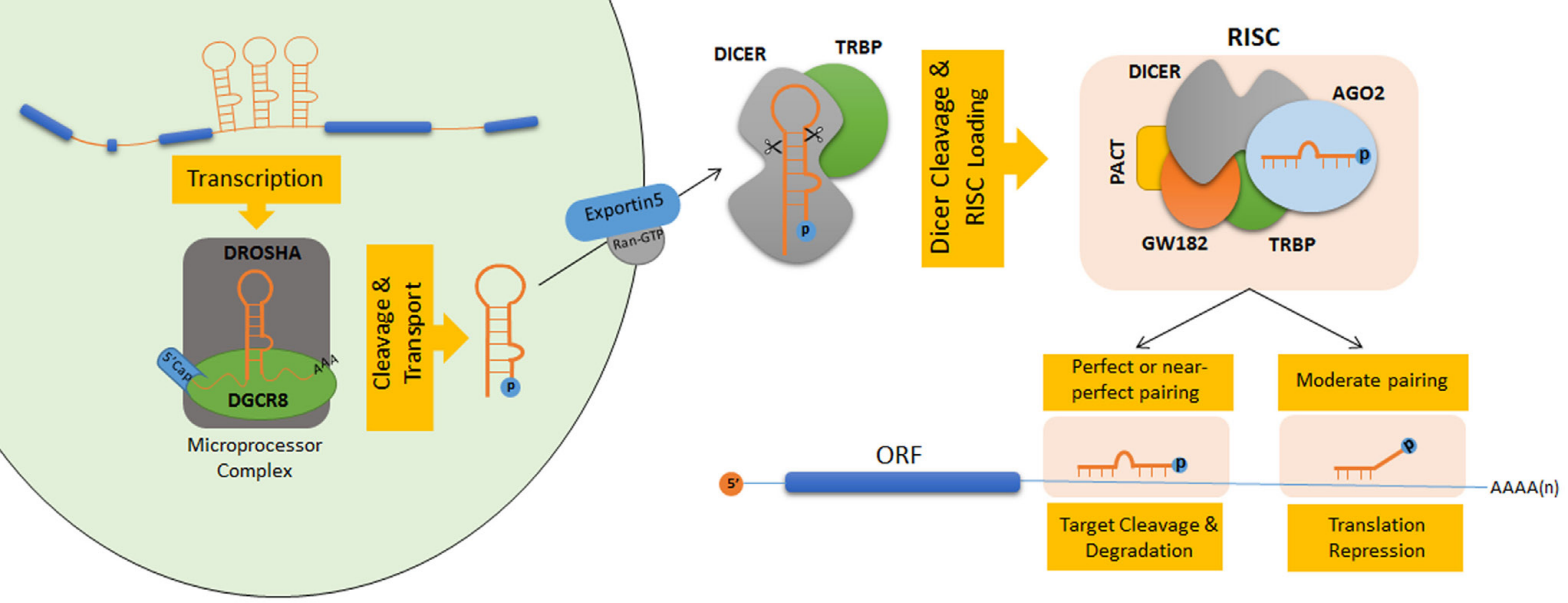
**Schematic model of microRNA (miRNA) biogenesis and maturation in human**. Nuclear cleavage events performed by protein complexes showing ribonuclease III activity lead to the processing of pri-miRNAs into small hairpin-shaped pre-miRNAs (22, 23). The core ribonuclease complex (the microprocessor complex) consists of a heterotetramer of Drosha and DGCR8 (DiGeorge syndrome critical region gene 8 or Pasha) proteins. During this reaction, flanking ssRNA–dsRNA junctions in pri-miRNAs are recognized by DGCR8 which guides Drosha to specific cleavage sites around 11 bp away from the stem–ssRNA junctions (19, 24). Pre-miRNAs that are released after Drosha cleavage exhibit characteristic features of RNase III endonuclease products having 5′ phosphate groups and 2 nt overhangs at their 3′ sequences (25). After cleavage by Drosha, 3′ overhangs are recognized by exportin-5 (XPO5) complexes in the nucleus (26). Pre-miRNAs are then transferred to the cytosol by canonical Ran-GTP-dependent transport mechanisms (27). In the cytosol, another RNase III-type endonuclease, called the Dicer, cleaves pre-miRNAs near their terminal loops, and leads to their conversion to double-stranded 20–22 nt miRNA duplexes (28–30). Terminal loop of pre-miRNAs are recognized through the N-terminal helicase domain of Dicer. Its PAZ domain interacts with 2 nt 3′ overhangs at the termini of pre-miRNAs and directs them to its catalytic RNase III domain for cleavage (30). RNA-induced silencing complex (RISC) captures the cleavage product through its Argonaute (AGO) protein component (31). ATP-dependent chaperone activity of Hsc70/Hsp90 proteins is important for small RNA duplex loading onto Ago proteins. Following passenger-strand degradation or ejection, AGO proteins remain in complex with a single-strand guide miRNA (32). In humans, among the four AGO proteins (AGO1–4), only the AGO2 protein has the ability to slice target mRNAs (33).

Following transcription, a number of consecutive RNAse-dependent reactions are required in order to process intermediary RNA oligonucleotides and produce mature and functional miRNAs. They are then processed in the nucleus by a core ribonuclease complex including Drosha and its regulatory subunit DGCR8 to generate hairpin-structured premature-miRNAs (pre-miRNAs) of 60–70 nt. After cleavage they can be recognized by exportin-5 and transport from nucleus to the cytoplasm. In cytoplasm, DICER protein further cleaves the hairpin structure of pre-miRNAs which leads to the formation of ~21–22 nt long miRNA duplexes. Then these duplexes loaded onto a complex called RNA-induced silencing complex (RISC). Argonaute (AGO) proteins are important components of the RISC complex which they guide single-stranded mature miRNAs to their target mRNAs. The fate of the mRNA determined the degree of complementarity between mature miRNA seed sequences (~8 nt in the core region of the miRNA) and “miRNA response elements (MRE)” on target mRNA sequences. In the slicer-dependent mechanism, base pairing with the guide miRNA results in an endonuclease-dependent cleavage of the target mRNA. miRNA-directed de-capping and/orde-adenylation of the target mRNA may proceed the degradation process whereas a partial complementarity may block the translation machinery (17, 21) (Figure 1).

### Autophagy

**Figure 2:**
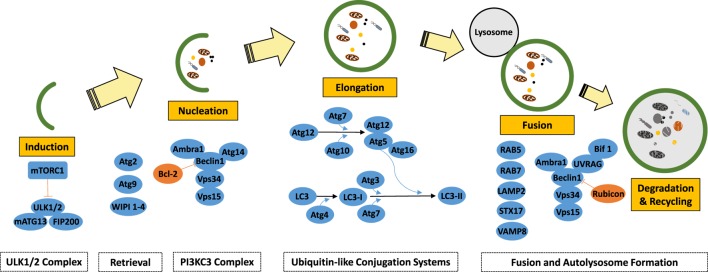
**Schematic representation of the autophagy pathway and core autophagy proteins**.

Macroautophagy (autophagy herein) is an evolutionary conserved cellular recycling pathway during which cargos, including long-lived proteins, protein aggregates, and damaged organelles (such as mitochondria and peroxisomes) are eliminated through lysosomal degradation. During this biological process, cargo molecules in the cytosol are sequestered by vesicles (autophagosomes) that are bound by double or multiple membrane bilayers (Figure 2) (34). Autophagosomes eventually fuse with lysosomes and form “autolysosomes,” leading to degradation of autophagic components and their cargos by the action of lysosomal hydrolases. Eventually following breakdown, degradation products are recycled back to cytosol, allowing their reuse by the cell. Basal autophagy is active in all eukaryotic cells and can be upregulated under a variety of cellular stress conditions, including starvation, growth factor deprivation, disease-related aggregate-prone protein accumulation, hypoxia, pathogens, etc.

Under stress, autophagy acts as a survival mechanism. It exerts a cytoprotective effect by providing building blocks and energy resources to cells, and by eliminating reactive oxygen generating damaged organelles and protein aggregates. These responses ensure adaptation to stress, promote energy homeostasis, and hence survival of the cell. On the other hand, under some conditions, uncontrolled autophagy was shown to lead to a caspase-independent, necrotic-like cell death that was called “the autophagic cell death.”

## Mechanisms of Autophagy

Autophagy is regulated by a set of evolutionarily conserved ATG gene products (35). In addition to ATGs proteins, several other proteins were implicated in autophagy regulation. miRNAs have been shown to regulate autophagy through their effects on various autophagy regulatory proteins that function at different stages of the pathway, namely induction, vesicle nucleation, vesicle elongation, retrieval, and fusion stages. Here, we will briefly overview autophagosome formation stages and introduce major proteins and complexes involved in the process (Figure 2). For a more detailed analysis, the readers are referred to the following excellent review articles (35, 36).

### Induction

Target of rapamycin (TOR) kinase-containing protein complexes are key regulators of the autophagy pathway. Mammalian TOR (mTOR) kinase forms two autophagy-related protein complexes: mTORC1 and mTORC2 complexes. In addition to mTOR, mTORC1 is composed of RAPTOR, GβL, and PRAS40 proteins. On the other hand, mTORC2 include mTOR, RICTOR, GβL, SIN1, and PROTOR proteins.

Various stress-causing signals, including amino acid starvation, growth factor deprivation, and low ATP levels, that are conveyed by RAG proteins, the AKT pathway, and the AMPK pathway, converge at mTORC1, that strictly coordinates cell growth-related events, including initiation of translation, ribosome biogenesis, protein synthesis, and cell size in the light of these inputs. On the other hand, mTORC2 is mainly involved in cytoskeletal reorganization and cell migration. The mTORC1 complex is also a major regulator of autophagy, yet mTORC2 also contributes to the control of autophagy through AKT pathway regulation.

mTORC1 is a central regulator of autophagy. Under normal conditions, mTORC1 keeps autophagy under control through direct inactivation of the ULK1/2 protein complex that is composed of ULK1/2, ATG13, ATG101, and FIP200 proteins (35). Stress triggers inactivation of the mTORC1 complex. ULK1/2 then can autophosphorylate and phosphorylate ATG13 and FIP200 proteins, and turn on the autophagosome initiation and nucleation machinery (37).

### Vesicle Nucleation

The next step in the canonical autophagy machinery is vesicle nucleation. It is initiated by the Class III phosphatidylinositol 3-kinase (PI3K) complex, consisting of the PI3K protein VPS34 and VPS30, ATG14/Barkor, VPS15, and ATG6/*BECN1* (Beclin 1) proteins. Lipid kinase activity of the PI3K complex is responsible from the accumulation of phosphatidylinositol 3-phosphate (PI3P) molecules on membranes, including the outer leaflet of the endoplasmic reticulum (ER). PI3P molecules serve as landing pads for autophagy-related proteins such as WIPI1–4 and DFCP1, marking sites of autophagosome formation and leading to omegasome/cradle development. Other regulators of the complex include BCL-2 family proteins, AMBRA1, and RUBICON (38).

### Elongation

Two ubiquitylation-like conjugation systems, namely the ATG12–ATG5–ATG16 and ATG8 (MAP1LC3, or briefly LC3 in mammals) systems, regulate autophagic membrane elongation and completion (35). The first system involves conjugation of the ATG12 protein to ATG5 through action of the E1-like enzyme ATG7 and the E2-like enzyme ATG10. The ATG12–ATG5 conjugate forms a larger multimeric complex of around 800 kDa in mammals with the addition of the ATG16L1 protein. The second system involves conjugation of the LC3/ATG8 protein to a lipid molecule, generally to a phosphatidylethanolamine (PE). LC3 should be cleaved at its carboxy terminus by ATG4 cysteine proteases in order to generate the cytosolic free LC3-I form that is capable of lipid conjugation. ATG7 (E1-like) and ATG3 (E2-like) proteins, as well as the ATG12–ATG5–ATG16L1 complex (E3-like activity) are required for the conjugation of PE to free LC3-I proteins, giving rise to autophagic membrane-bound LC3-II form. By this way, LC3 proteins ensure elongation and expansion of autophagic membranes and their closure.

### ATG9-Dependent Vesicle Retrieval

ATG9 (mammalian homolog: ATG9L1) is a multi-spanning transmembrane protein that localizes not only to PAS but also to endosomes and to the trans-Golgi network (39).

Cycling between these compartments, ATG9 is necessary for lipid delivery to autophagosomes and recycling of some proteins. ATG9 trafficking is regulated by RAB proteins (e.g., RAB1 and RAB11), TRAPP protein complexes, ATG2 and ATG18 (mammalian WIPI1–4) proteins (40, 41).

### Lysosomal Fusion

At the final stage, outer membrane of mature autophagosomes fuses with lysosomal membranes to form autolysosomes (42). Autophagosome–lysosome fusion machinery involves SNARE complexes (e.g., VAMP8, STX17), integral lysosomal proteins (e.g., LAMP2), and RAB proteins (e.g., RAB5 and RAB7). Moreover, dyneins are necessary for the transport of autophagosomes along microtubules, allowing them to meet late endosomes and lysosomes. BIF1 and UVRAG proteins that play a role in the regulation of membrane curvature formation and endosomal trafficking contribute to the formation and maturation of autophagosomes through their interaction with the *BECN1*/Beclin 1 protein (43, 44). Following fusion, cargos that are carried by autophagosomes are digested by lysosomal acid hydrolases, including cathepsins, and they are broken down to their building blocks (e.g., proteins into amino acids). Recycling of digested molecules is achieved following their transport from lysosome lumen into the cytosol.

### Autophagy Receptors

Historically, autophagy described as a non-selective important cellular homeostasis phenomenon (45). However, identification of different autophagy receptors that are able to recognize different cargos pointed out to the selectivity of the autophagy (46, 47). A number of autophagy receptors have been discovered including SQSTM1/p62 (48), NBR1 (49), NDP52 (also known as a CALCOCO2) (50), OPTN (51), and NIX (also known as BNIP3L) (52). These receptors share motifs that allow bridging between LC3 on the autophagosomes [LC3-interacting region (LIR)] and generally ubiquitylated targets [ubiquitin-binding domain (UBD)]. Since they are also delivered to autolysosomes together with the cargo, autophagy receptor degradation is commonly used as a marker of autophagic degradation activity.

## Interplay Between miRNAs and Autophagy

### Regulation of Autophagy by miRNAs

Studies in recent years introduced miRNAs as new players in the regulation of autophagy. Indeed, miRNAs were shown to change levels of several key proteins that are playing a role at various steps of the autophagy pathway, from upstream signaling pathways to later stages of autolysosomal degradation.

### Regulation of Induction by miRNAs

As major upstream regulators of the autophagy pathway, mTOR-containing protein complexes and other proteins in the pathway were shown to be direct or indirect targets of a number of miRNAs. For example, five different components of the mTOR pathway, namely p70S6K, eukaryotic translation initiation factor 4E (eIF4E), Mknk1, Mknk2, and Mapkap1, were identified as direct targets of miR-7.

In hepatocellular carcinoma (HCC) cells, miR-7 was also introduced as a key regulator of the PI3K/Akt pathway, and shown to target mTOR, p70S6K, and PIK3CD (53). miR-199a and miR-101 were other miRNAs that could directly target mTOR in different cell types (54–57).

ULK1/2 complex components were also direct targets of miRNAs. In squamous cell carcinoma cells, cisplatin-induced miR-885-3p directly targeted ULK2 and contributed to the regulation of autophagy (58).

miR-26b targeted ULK2 as well, inhibiting autophagy in prostate cancer cells (59). Direct interactions between *MIR7* cluster members miR-20A and miR-106b and ULK1 could lead to the inhibition of leucine deprivation-induced autophagy in C2C12 myoblast cells, while blockage of endogenous miR-20a and miR-106b could restore normal autophagic activity (60). In another study, miR-25 was introduced as a novel regulator of autophagy and cell death through its direct effects on ULK1 expression (61). miR-17*-*5p, an miRNA that was upregulated upon BCG infection of macrophages, also regulated ULK1. By this way, miR-17*-*5p inhibited host cell autophagy that could normally eliminate intracellular BCG (62). In another study, Chen and coworkers proposed that ULK1 could inhibit p70S6K in starvation-induced autophagy of neuroblastoma SH-SY5Y cells and further identified that miR-4487 and miR-595 were novel ULK1-targeting miRNAs (63).

### Regulation of Vesicle Nucleation by miRNAs

miR-30a was among the first miRNAs to be implicated in autophagy regulation. Zhu et al. showed that miR-30a inhibited rapamycin-induced autophagy in MCF-7 cells by directly targeting *BECN1*/Beclin 1 (64). Autophagy regulation by miR-30a was confirmed in subsequent studies. miR-30a sensitized HeLa cells to chemotherapy, through attenuation of cisplatin-induced autophagy in a *BECN1*/Beclin 1-dependent manner. Moreover, combined treatment with imatinib and miR-30a increased drug sensitivity in chronic myeloid leukemia cells through regulation of ATG5 and *BECN1*/Beclin 1 (65). In line with these data, a recent study showed that miR-30a levels were significantly reduced in chemoresistant osteosarcoma cells (66).

In a functional unbiased miRNA screen, we have found that *BECN1*/Beclin 1 could be targeted by the members of the *MIR376* family. miR-376a and miR-376b regulated starvation- and rapamycin-induced autophagy in breast and liver cancer cells by directly targeting 3′-UTR sequences of *BECN1*/Beclin 1 and ATG4C (67, 68). Moreover, these studies led us to propose “a gas and break model” of autophagy regulation under stress conditions: According to this model that was supported by our experimental data, autophagy activating stress signals trigger sequential upregulation of autophagy inhibitory miRNAs, and miRNA-mediated limitation of the autophagic activity prevents hyperactivation of autophagic degradation and ensures survival during prolonged stress conditions (68, 69).

In addition to *MIR30A* and *MIR376* family members, *BECN1*/Beclin 1 was identified as a target of miR-519a as well. In squamous cell carcinoma cells, miR-519a was shown to block autophagy that was activated by cisplatin (58). On the other hand, irradiation-induced autophagy in breast cancer cells was controlled by miR-199*-*5p that downregulated both *BECN1*/Beclin 1 and DRAM1 (70). In another study, high-fat diet upregulated miR-384*-*5p in a mouse model of atherosclerosis, and this miRNA impaired macrophage autophagy through direct targeting of *BECN1*/Beclin 1. In this context, autophagy deficiency in macrophages further promoted development of atherosclerosis (71). In colon cancer cells, oxaliplatin-induced autophagy was inhibited by miR-409*-*3p (72). Here, miR-409*-*3p-targeted *BECN1*/Beclin 1 and sensitized tumor cells to chemotherapy. Another miRNA that suppressed *BECN1*/Beclin 1 was miR-216a. Irradiation-mediated autophagy was blocked, and apoptosis was activated in radioresistant pancreatic cancer cells through action of this miRNA (73).

Other regulators of the *BECN1*/Beclin 1-VPS34 complex were also modulated by miRNAs. AMBRA1 was identified as an miR-23a target in dermal human fibroblasts which were exposed to either UVB or PUVA irradiation, and miR-23A-specific antagomirs increased autophagy (74).

### Regulation of Elongation by miRNAs

Components of autophagy-related ubiquitination-like conjugation were also shown to be controlled by miRNAs. Independent studies showed that miR-181a, miR-30a, miR-374a, and miR-224*-*3p could directly target ATG5, miR-30d, miR-630, and miR-200b suppressed ATG12 while miR-20a and miR-885*-*3p targeted Atg16L1, and miR-519A could affect levels of both ATG16 and ATG10 (58, 65, 75–80).

A number of studies in the literature provided evidence that ATG7 levels were regulated by several different miRNAs. Suppression of autophagy through targeting of ATG7 by miR-375 was shown to reduce viability of HCC cells during hypoxia (81). ATG7 was also a target of miR-20a that also affected ATG16L1 levels (82). Another miRNA that was shown to target ATG7 was miR-17. The miRNA could modulate autophagy by negatively regulating ATG7 expression in human glioblastoma cells (83). Moreover, miR-137, which takes part in neuronal maturation and neurogenesis, suppressed starvation-induced autophagy by targeting ATG7 in glioblastoma cells (84). Another study revealed that under hypoxia stress, miR-96 played a dual role in autophagy regulation in prostate cancer cells. miR-96 could fine tune autophagy by targeting ATG7 and mTOR (85). In another study, Wang et al. showed that miR-188*-*3p could specifically participate in the regulation of ATG7 expression and impair autophagy in the heart (86). Ectopic expression of miR-199a-5p decreased ATG7 protein levels and suppressed autophagy in HCC cells (87).

Both LC3 and LC3 processor ATG4 family members were regulated by miRNAs. Another study showed an indirect correlation between miR-204 and LC3 levels. Upregulation of miR-204 levels upon myocardial ischemia-reperfusion caused an increase in LC3 protein levels in adult rat models (88). In addition to *BECN1*/Beclin 1, miR-376 family members miR-376a and miR-376b could negatively regulate ATG4C in breast and liver cancer cells (67, 68). In a luciferase-based functional miRNA screen, another member of ATG4 family, ATG4D, was identified as a target of miR-101. The same miRNA was introduced as an inhibitor of basal as well as rapamycin- and etoposide-induced autophagy (89). *SQSTM*1*/p*62 encoding for a selective autophagy receptor was reported to be directly targeted by the *MIR17/20/93/106* family of miRNAs (90).

### Regulation of ATG9-Dependent Retrieval by miRNAs

miR-34a was shown to regulate ATG9A levels during angiotensin II-induced myocardial hypertrophy (5) as well as during neural stem cell differentiation (91). Another protein in the same pathway, the ATG2B was identified as a direct target of miR-130a, an miRNA that inhibited autophagy and promoted cell death in chronic lymphocytic leukemia cells (92). ATG2 was also among the targets of miR-30D, an miRNA that was shown to target multiple core proteins in the autophagy pathway (78).

### Regulation of Autophagosome Maturation and Lysosome Fusion by miRNAs

A number of miRNAs were reported to control the autophagosome–lysosome fusion machinery as well. RAB proteins regulating endocytic pathways namely RAB1B, RAB22A, RAB14 were shown to be targeted by miR-502, miR-373, and miR-451, respectively (93, 94). Furthermore, miR-205 could downregulate lysosomal proteins RAB27A and LAMP3 in a prostate cancer cell model (95). In a study involving ischemic injury and spontaneous recovery, LAMP2 was identified as a direct target of miR-207 (96). miR-487*-*5p could target LAMP2 as well (97). UVRAG that also plays a role in endosomal trafficking and autophagosome maturation was shown to be a target of miR-374, miR-630, miR-125, and miR-351 that have and inhibitory effect on autophagy (76, 98).

### Control of miRNAs and miRNA Generation Pathways by Autophagy

A complex interplay between the autophagy machinery and miRNA biogenesis and maturation systems exists. miRNA-processing enzymes, DICER1, and the RISC component AGO2 were described as direct targets of autophagolysosomal degradation. In fact, DICER1 and AGO2 were found to associate with the autophagy receptor NDP52 in a GEMIN3/4-dependent manner, and receptor–target complexes were degraded upon autophagy activation (99). On the other hand, downregulation of DICER1 expression attenuated autophagy induction during acute promyelocytic leukemia differentiation (100). Similarly, targeting of ATG2B and DICER1 by miR-130A inhibited autophagy in chronic lymphocytic leukemia cells, and knockdown of DICER1 alone was sufficient to block autophagy in this context (92). In line with these findings, AGO2 accumulation was observed in ATG5^−/−^ and ATG16^−/−^ mouse embryonic fibroblasts and ATG7^−/−^ intestinal organoids (101). Although it was suggested that autophagy degraded only small RNA-free DICER1 and AGO2 proteins, degradation of a number of miRNAs including miR-224 was reported to be dependent on the autophagic activity (102). All these data indicate that miRNA-autophagy connections work both ways. Therefore, regulation of autophagy by miRNAs might have cellular consequences beyond mere autophagy suppression or activation, potentially having repercussions on miRNA control pathways and global miRNA landscape in cells.

## Autophagy and Cancer

### Autophagy As a Tumor Suppressor

Studies in the literature draw a complex picture about the involvement of autophagy in cancer formation and progression. The role of autophagy seems to be context- and tumor type-dependent, i.e., early versus late stage disease, fast versus slow growing tumors show different degrees of autophagy dependence.

Studies focusing on early stages of cancer formation indicate a tumor-suppressor role of autophagy during malignant transformation. For example, haploinsufficiency of *Becn*1*/Beclin* 1 in genetically modified mice resulted in tumor formation in various systems, including lung adenocarcinomas, HCCs, and heamatological malignancies (103–107). Similarly, *Atg*5 and *Atg*7 deletions in the liver resulted in the formation of liver adenomas (108). *Atg*4C*-*deficient mice were prone to develop fibrosarcomas that were induced by chemical carcinogens (109). In line with these results, *UVRAG* expression suppressed and *Bif*1 deletion enhanced tumor formation in mice (43, 44). Analysis of a series of human tumors confirmed these experimental results. For example, monoallelic deletions and lower *BECN1* protein levels were found in human prostate, breast, and ovarian cancers tissues that were analyzed (103, 104). Similarly, *ATG*5 expression was lost in human gastric, colorectal, and HCC specimen, and monoallelic mutations of UVRAG were reported to be frequent in human colon cancers (110–112). Mechanisms of cancer suppression by autophagy were studied as well. Autophagy is responsible for the degradation of abnormally folded and/or mutant proteins and damaged organelles (e.g., mitochondria) that in fact constitute a major source of reactive oxygen species (ROS). Consequently, elimination of these sources of ROS by autophagy was shown to alleviate DNA damage accumulation and prevent genomic instability (11). Targeted elimination of some cancer-related proteins by autophagy was also reported. Autophagy-dependent selective degradation of oncogenic SQSTM1 (P62), PML-RARA, mutant p53, and BCR-ABL1 proteins may be cited as prominent examples (113–116). Autophagic degradation of hypoxia-inducible and proangiogenic HIF2α protein in a constitutive manner was also reported to suppresses kidney tumorigenesis (117).

Moreover, while autophagy mainly acts as a prosurvival mechanism and a stress response, autophagy activation under certain conditions was connected to cell death (118–120). Hence at least in some contexts, autophagic cell death might also contribute to tumor suppressive functions. In line with this view, blockage of autophagy in some contexts prevented death of cancer cells [e.g., Ref. (121, 122)]. Furthermore, several tumor suppressor and death-related proteins, including DAPK, DRP1, ZIPk, and a p19ARF form (smARF) triggered a non-apoptotic and autophagy-dependent cell death in cancer cells (123–126). Oncogene-induced senescence that eventually leads to cell death was also shown to depend on autophagy (127). On the other hand, same hostile conditions (e.g., starvation and low nutrient supplies) that trigger autophagy may also activate phenomena such as entosis (cell-in-cell) where cancer cells cannibalize each other [and the references therein; (128, 129)].

The role of autophagy in immune responses and inflammation was also suggested to be important for its cancer-related effects. For instance, anticancer immunosurveillance that involves recognition and elimination of nascent cancerous cells by the immune system may be dually regulated by autophagy in different cell types (e.g., development and maturation of immune system components versus hijacking of the immune system by tumor autophagy) (130, 131). Additionally, autophagy was shown to limit inflammation that, in especially in a chronic form, is a major trigger form of some types of cancer (e.g., HCC). Elimination of inflammasomes and limitation of pro-inflammatory interleukins (132, 133) and NF-kB signaling (134) as well as inhibition of pro-inflammatory signals controlled by pattern recognition receptors (135, 136) and prevention metabolic stress and inflammatory cell infiltration to tissues (137) all depended on intact autophagy function.

### Autophagy As a Tumor Promoter

In established and especially fast-growing tumors, survival-related role of autophagy predominates. Cancer cells face with unfavorable conditions that challenge their endurance to various types of stress. Abnormal and insufficient tumor vascularization leads to hypoxia, changes in local pH, scarce nutrient, growth factor, and hormone supply, while energy and oxygen demands increase due to fast proliferation. Therefore, the tumor environment imposes high levels of metabolic stress upon malignant cells. Autophagy supports tumor cell survival and growth under these harsh conditions. For example, in oncogenic RAS- or RAF-driven fast-growing tumors, autophagy ensured tumor cell proliferation and survival, mitochondrial quality control and maintenance of energy levels, building block (e.g., aminoacid) abundance. These autophagy-dependent conditions were crucial factors supporting metabolic activities of cancer cells (9, 138). Elevation of basal autophagy levels was especially indispensable for the survival of tumor cells that were found in the less vascularized regions of solid tumors (137).

Cells from invasive and metastatic tumors are subject to extreme stress that originates from detachment from neighboring cells and from the basal lamina in their tissue of origin, evasion from the primary sites, shear forces and immune system attack in the blood stream, invasion and spread in a “foreign” secondary site (139). Under these conditions, autophagy was shown to provide resistance to metabolic stress conditions and anoikis (detachment-induced cell death) supporting cancer cell survival (9, 140–142). Autophagic capacity of tumor cells was reported as a determining factor during epithelial–mesenchymal transition (EMT), metastasis, and dormancy of tumor cells in some contexts (143, 144). Yet in HCC cells, EMT and migration properties were not affected, but anoikis resistance and distant metastasis capacity were reduced when autophagy was suppressed (145). In another study, knockdown of ATG5 in melanoma cells decreased cells’ capacity to survive metabolic stress and to colonize lungs in mice following intravenous injection (146). Similarly, depletion of ATG12 decreased the invasive capacity of glioma cells (147).

Furthermore, motility, invasion, and metastatic capacity of oncogenic RAS-transformed tumor cells depended on their autophagy competence and autophagy-dependent production of secreted factors (141). Establishment of dormancy state and survival of dormant cancer cells depended on their autophagy competence. For example, induction of autophagy by ARH-I/DIRAS3 was essential for dormancy of ovarian cancer cell micro metastases in xenograft models (148).

Autophagy plays a critical role in endothelial cell biology as well as tumor vascularization. Although endothelium-specific deletion of the key autophagy gene Atg7 in mice did not result in any prominent vascular abnormality or vascular density change, there were abnormalities of endothelial cell function (e.g., defect in the maturation and secretion of von Willebrand factor) (149). In a cancer context, selective degradation of angiogenesis regulators such as gastrin-releasing peptide or HIF2α by autophagy affected tumor vasculature and limited tumor growth (117, 150). In line with these observations, *BECN1*/Beclin 1 heterozygous mice had higher levels of circulating erythropoietin and HIF2α, increased angiogenesis under hypoxia, and enhanced tumor growth compared to wild-type mice (151). ATG5 knockdown in B16-F10 melanoma cells increased tumor vessel tortuosity; on the other hand, endothelial cell-specific deletion of ATG5 led to the formation of smaller and less mature tumor vasculature with endothelial cell lining and perfusion defects (146). Therefore, autophagic activity is important for angiogenesis under physiological and pathological conditions.

Cancer metabolism found to be distinct from that of normal healthy cells. High metabolic demands drive cancer cells to evolve different strategies such as usage of glycolysis and other alternative metabolic pathways (e.g., salvage pathways) as sources of energy. These metabolic conditions in combination with the hypoxic environment that accompanies rapid tumor growth and poor vascularization usually result in acidosis. Acidic tumor microenvironment has also been found to alter autophagy cancer cells as an adaptation mechanism to rough environmental conditions (152–154).

### Autophagy and Cancer Treatment

An important response of cancer cells to treatment with anticancer agents and radiation is autophagy activation (155). In most cases, autophagy confers resistance to anticancer therapy, yet in some tumor types, activation of autophagy was reported to have lethal effects on cancer cells. In any case, strategies aiming at modulation of autophagy bear the potential of improving responses to classical anticancer agents. Choice of the best strategy seems to depend on tumor type as well as tumor stage and treatment type. Additionally, autophagy manipulation renders otherwise resistant cancer types sensitive to therapeutic agents, and combination of autophagy drugs with conventional treatments might overcome drug resistance (156).

Sensitization to chemotherapy is one of the most studied topics in the autophagy field. In the scientific literature, beneficial effects of the combination of autophagy modulators with chemotherapy or radiotherapy were extensively studied. In many cancer types, inclusion of PI3K inhibitors (e.g., 3-MA or LY294002) in experimental treatments enhanced the efficacy of various chemotherapeutic agents and radiation through their autophagy blocking effects. For example, treatment with 3-MA sensitized esophageal squamous carcinoma cells to radiation therapy (157). Similarly, administration of 3-MA enhanced the efficacy of 5-Fluorouracil and cisplatin and promoted apoptosis in colon and lung cancer cells (158, 159). On the other hand, lysosomotropic agents [e.g., Chloroquine (CQ) or hydroychloroquine (HQ)] that neutralize the pH of lysosomes and that prevent autolysosome formation were shown to exert anticancer effects and/or enhance the efficacy of antineoplastic treatments in numerous publications [e.g., Ref. (160–162)]. For instance, in non-small-cell lung cancer bevacizumab plus CQ combination was found to increase the efficacy of cancer treatment (161).

Concomitantly, CQ and HCQ potentiated cytotoxic effects of p53 and alkylating agents in a mouse model of lymphoma (163). siRNA-based depletion of autophagy modulators was also able to sensitize carcinoma cells from different origins to chemotherapy and radiation treatment (164).

## Autophagy, miRNAs, and Cancer

Among autophagy-related miRNAs, many of them were involved in different stages of cancer formation and progression. These miRNAs were shown to influence cancer growth, cancer cell metabolism, hypoxia responses and neovascularization, cancer cell migration, and metastasis, and even response to drugs and radiotherapy. Moreover, some autophagy-related miRNAs were tested as anticancer agents or cancer biomarkers. In many studies, it was suggested that the effects of miRNAs on autophagy genes and proteins were critical for cancer-related outcomes, but in others data were correlative. Conversely in some other cases, targeting of miRNAs or miRNA-related components by autophagic degradation systems were decisive in the control of cancer progression. In this section, we will summarize existing literature that mainly implicates autophagy-related roles of these miRNAs in cancer biology and clinical outcomes (see Table 1 for a complete list of miRNAs).

**Table 1:** **Autophagy-related microRNAs (miRNAs) in cancer**.

miRNAs	miRNA status in cancer	Effect on autophagy	Autophagy-related targets	Type of target interaction	Tested cell line (tissue origin)	Reference
miR-let7f1	N.D.	Inhibition	HMGB1	Direct	UW228, D425 (medullablastoma)	(165)
miR-7	N.D.	Activation	EGFR	Indirect	H1299, A549 (lung cancer) T.Tn (esophageous)	(166)
miR-7	N.D.	N.D.	PIK3CD, mammalian TOR (mTOR) p70S6K	Direct	QGY-7703 (hepatacellular carcinoma)	(53)
miR-9*-*3p	N.D.	Inhibition	ATG5	Direct	TT and MZ-CRC-1 (medullary thyroid carcinoma)	(167)
miR-10a	Upregulated	Inhibition	Bim, TFAP2C, p16, and p21	Direct	U251, LN-308, and U373 (glioblastoma)	(168)
miR-15a*/*16	N.D.	Activation	RICTOR	Direct	HeLa (cervical cancer)	(169)
miR-16	Downregulated	Inhibition	BCL-2	Direct	A549-T24 (lung cancer)	(170)
miR-17						
miR-17, miR-19b miR-20a	N.D.	Activation	PTEN, PTENP1	Indirect	Mahlavu (hepatacellular carcinoma)	(171)
miR-17	Upregulated	Inhibition	ATG7	Direct	T98G and U373-MG (glioblastoma)	(83)
miR-17*-*5p	Downregulated	Inhibition	BECN1	Direct	A549-T24 (lung cancer)	(172)
miR-18a	N.D.	N.D.	hnRNPA1	Indirect	SW620 and HCT116 (colorectal cancer)	(173)
miR-20a	Upregulated	N.D.	ATG7	Direct	SiHa (cervical cancer)	(82)
miR-20a	Downregulated	Inhibition	FIP200	Direct	MCF-7, MDA-MB-231 (breast cancer)	(174)
miR-20a						
miR-21	Upregulated	Inhibition	N.D.	Indirect	U373, U87 (glioblastoma)	(175)
miR-21	N.D.	Inhibition	PTEN	Direct	SiHa, HeLa (cervical cancer)	(176)
miR-21	Upregulated	Inhibition	PTEN	Indirect	SiHa, HeLa (cervical cancer)	(177)
miR-21	Upregulated	Inhibition	PTEN	Indirect	Huh7, HepG2 (liver cancer)	(178)
miR-21	N.D.	Inhibition	PTEN	Indirect	MCF-7 (breast cancer)	(179)
miR-21	Upregulated	N.D.	PDCD4	Indirect	Tca8113 (tongue squamous cell carcinoma)	(180)
miR-22	N.D.	Inhibition	HMGB1	Direct	MG-63 (osteosarcoma)	(181)
miR-22	N.D.	Inhibition	BTG1	Direct	SW620, RKO (colorectal cancer)	(182)
miR-23a	Upregulated	N.D.	TOP2B	Indirect	Tca8113 (tongue squamous cell carcinoma)	(183)
miR-23a	Downregulated	Inhibition	ATG12	Direct	BxPC3 (pancreas)	(184)
miR-23B*-*3p	Downregulated	Inhibition	HMGB2, ATG12	Direct	SGC7901/VCR (gastric cancer)	(185)
miR-24*-*3p	Downregulated	Inhibition	ATG4A	Direct	H446/EP (lung cancer)	(80)
miR-25	N.D.	Inhibition	ULK1	Direct	MCF-7 (breast cancer)	(61)
miR-26a	Downregulated	Inhibition	ULK2	Direct	PC3, C4-2 (prostate cancer)	(59)
miR-29a	N.D.	N.D.	HDAC4	Direct	KMS11, SKMM1, and NCI-H929 (myeloma)	(186)
miR-29b	Downregulated	Inhibition	PSME4	Direct	AMCL1, AMCL2 (myeloma)	(187)
miR-30a	N.D.	Inhibition	BECN1	Direct	MDA-MB-468, MCF-7 (breast cancer) H1299 (lung cancer) T98G (glioblastoma)	(64)
miR-30a	N.D.	Inhibition	BECN1, ATG5	Direct	K562 (CML)	(65)
miR-30a	N.D.	Inhibition	BECN1	Indirect	HeLa (cervical cancer)	(188)
miR-30a	Downregulated	Inhibition	BECN1	Indirect	786-0, A489 (renal carcinoma)	(189)
miR-30a	Downregulated	Inhibition	BECN1	Direct	MG-63 (osteosarcoma)	(66)
miR-30a	Downregulated	Inhibition	BECN1	Indirect	SH-SY5Y (neuroblastoma)	(190)
miR-30d	N.D.	Inhibition	ATG2B, ATG12, ATG5, BNIP3L	Direct	A2780, OVCAR10 and 2008 (ovarian cancer), T47D and MCF-7 (breast cancer)	(78)
miR-30d	N.D.	Inhibition	BECN1	Direct	SW1736, 8305 C (anaplastic tyroid carcinoma)	(191)
miR-32	N.D.	Inhibition	DAB2IP	Direct	PC3, DU145 (prostate)	(192)
miR-34a	N.D.	Inhibition	HMGB1	Direct	Y79, Weri-RB1 (retinoblastoma)	(193)
miR-34*-*5p miR-5195*-*3p	Upregulated	N.D.	BECN1	Direct	A172, T98G (glioblastoma)	(194)
miR-93*/*106b	N.D.	Activation	p21	Indirect	SaOS-2 and MNNG/HOS (osteosarcoma)	(195)
miR-96	N.D.	Bi-phasic regulation	mTOR, ATG7	Direct	LNCaP, 22Rv1, and LAPC4 (prostate cancer)	(85)
miR-100	Downregulated	Activation	IGFR1, mTOR	Direct	HepG2, Huh7 (liver cancer)	(196)
miR-101	N.D.	Inhibition	STMN1, ATG4D, RAB5A	Direct	MCF-7 (breast cancer)	(89)
miR-101	N.D.	Inhibition	STMN1, ATG4D, RAB5A, mTOR	Direct	HepG2 (liver cancer)	(197)
miR-101	Downregulated	Inhibition	EZH2	Direct	HepG2 (liver cancer)	(198)
miR-101	Downregulated	Inhibited	STMN1	Direct	CNE-2, 5–8 F, and 6-10B (nasopharyngeal carcinoma)	(199)
miR-106	N.D.	Inhibition	ATG16L1	Direct	HCT116 (colorectal cancer)	(200)
miR-93						
miR-124	Downregulated	Activation	PTB1	Direct	DLD-1, WiDr (colorectal cancer)	(201)
miR-124	Downregulated	Inhibition	PIM1	Direct	DU145 and PC3 (prostate cancer)	(202)
miR-144						
miR-125b1	Upregulated	Inhibition	DRAM2, ATG4D UVRAG	Direct	NB4 (acute promyelocytic leukemia)	(203)
miR-126	Downregulated	Activation	IRS1	Indirect	Met5a, H28, and IstMes2 (malignant mesothelmia)	(204)
miR-129	N.D.	Activation	NOTCH1	Direct	U87, U231 (glioblastoma)	(205)
miR-130a	Downregulated	Inhibition	ATG2B, DICER1	Direct	MEC-1 (leukemia)	(92)
miR-137	N.D.	Inhibition	ATG7	Indirect	U87 (glioblastoma)	(84)
miR-138	N.D.	Activation	BIM	Direct	LN-308, ZH-305 (glioblastoma)	(206)
miR-140*-*5p	Downregulated	Inhibition	SMAD2	Direct	HCT116, RKO, and SW480 (colorectal cancer)	(207)
miR-143	Downregulated	Inhibition	GABARAPL1	Direct	AGS and MKN28 (gastric cancer)	(208)
miR-143	N.D.	Inhibition	ATG2B	Direct	H1299 (lung cancer)	(209)
miR-144	Downregulated	Activation	TIGAR	Direct	A549, H460 (lung cancer)	(210)
miR-152	Downregulated	Inhibition	ATG14	Direct	A2780/CP70, SKOV3/DDP (ovarian cancer)	(211)
miR-155	N.D.	Activation	RHEB, RICTOR RPS6KB2	Direct	NSE (nasopharyngeal cancer) and HeLa (cervical cancer)	(212)
miR-155*-*3p	N.D.	Activation	CREBRF	Direct	U251 and T98G (glioblastoma)	(213)
miR-181a	N.D.	Inhibition	ATG5	Direct	MCF-7 (breast cancer) Huh7 (liver cancer) K562 (chronic myelogenous leukemia)	(75)
miR-181a	N.D.	Inhibition	ATG5	Indirect	SGC7901/CDDP (gastric cancer)	(214)
miR-183	Upregulated	Inhibition	UVRAG	Indirect	HCT116 and HT29 (colorectal cancer)	(215)
miR-193b	Upregulated	Activation	STMN1	Indirect	KYSE450 (esophageal cancer)	(216)
miR-199a	N.D.	N.D.	mTOR	Direct	Huh7, HepG2, SNU475 (liver cancer)	(55)
miR-199A*-*5p	Downregulated	Inhibition	ATG7	Direct	Huh7, HepG2 (liver cancer)	(87)
miR-199A*-*5p	N.D.	Inhibition	DRAM1, BECN1	Direct	MCF-7, MDA-MB-231 (breast cancer)	(70)
miR-200b	N.D.	Inhibition	ATG12	Direct	SPC-A1/DTX, H1299/DTX (lung cancer)	(217)
miR-200c	N.D.	Activation	UBQLN1	Direct	MDA-MB-231 (breast cancer)	(218)
miR-204	N.D.	Inhibition	Transient receptor potential melastatin 3 (TRPM3)	Direct	786-O, A498, and Caki-1 (kidney cancer)	(219)
miR-204	N.D.	Inhibition	LC3	Direct	786-O, A498, and Caki-1 (kidney cancer)	(220)
miR-205	Downregulated	Inhibition	RAB27A, LAMP3	Indirect	DU145, PC3 (prostate cancer)	(95)
miR-205	N.D.	Inhibition	TP53INP1	Direct	DU145, LNCaP (prostate cancer)	(221)
miR-212	Downregulated	Inhibition	SIRT1	Direct	LnCap, PC3 (prostate cancer)	(222)
miR-214	Downregulated	Inhibition	UCP2	Direct	MCF-7/LCC9 (breast cancer)	(223)
miR-214	N.D.	Inhibition	LC3A, LC3B	Direct	786-O, A498, and Caki-1 (kidney cancer)	(219)
miR-216a	Upregulated	Inhibition	BECN1	Direct	PANC-1 (pancreas cancer)	(73)
miR-216b	Downregulated	Inhibition	BECN1	Direct	A549, Calu-3 (lung cancer)	(224)
miR-218	Downregulated	Inhibition	HMGB1	Direct	RL95-2 (endometrial carcinoma)	(225)
miR-224	Upregulated	Inhibition	SMAD4	Direct	Hep3B, Hbx transgenic mice (liver cancer)	(102)
miR-224*-*3p	Downregulated	Inhibition	ATG5, FIP200	Direct	U251 and U87 (glioblastoma)	(77)
miR-224*-*3p	Upregulated	Inhibition	FIP200	Direct	HeLa, SiHa, C33A (cervical cancer)	(226)
miR-290*-*295	N.D.	Inhibition	ULK1, ATG7	Direct	B16F1, R2L (melanoma)	(227)
miR-340	Downregulated	Inhibition	ROCK1	Direct	U373, U87 (glioblastoma)	(228)
miR-372	N.D.	Inhibition	SQSTM1	Direct	MCF-7, MCF10A (breast cancer)	(229)
miR-373	Downregulated	N.D.	RAB22A	Direct	SKOV3 (ovarian cancer)	(230)
miR-374a	N.D.	Inhibition	UVRAG, ATG5	Direct	JHU-029 (squamous cell carcinoma)	(76)
miR-375	Downregulated	Inhibition	ATG7	Direct	Huh7, Hep3B (liver cancer)	(81)
miR-376a	N.D.	Inhibition	BECN1,ATG4C	Direct	MCF-7 (breast cancer) Huh7 (liver cancer)	(68)
miR-376b	N.D.	Inhibition	BECN1, ATG4C	Direct	MCF-7 (breast cancer) Huh7 (liver cancer)	(67)
miR-409*-*3p	Downregulated	Inhibition	BECN1	Direct	Lovo Oxa R (colorectal cancer)	(72)
miR-451	Downregulated	N.D.	RAB14	Direct	A549, SPC-A1, and NCI-H520 (lung cancer)	(93)
miR-451a	N.D.	Inhibition	N.D.	N.D	MCF-7, LCC2 (breast cancer)	(231)
miR-487b-5p	Upregulated	Inhibition	LAMP2	Direct	A549, H1299 (lung cancer)	(97)
miR-502	Downregulated	Inhibition	RAB1B	Direct	HCT116 (colorectal cancer)	(94)
miR-519a	N.D.	Inhibition	BECN1, ATG10 ATG16L1	Direct	JHU-029 (squamous cell carcinoma)	(76)
miR-630	N.D.	Inhibition	ATG12, UVRAG	Direct	JHU-029 (squamous cell carcinoma)	(76)
miR-634	N.D.	Inhibition	XIAP, BIRC5, APIP, OPA1, TFAM, LAMP2	Direct	KYSE850 (esophageal squamous cell carcinoma)	(232)
miR-638	Upregulated	Inhibition	TP53INP2	Direct	SK-Mel-28 and SK-Mel-147 (melanoma)	(233)
miR-885*-*3p	N.D.	Inhibition	ULK2,AKT1,BCL-2 ATG16L2	Direct	JHU-029 (squamous cell carcinoma)	(58)
miR-4487	N.D.	Inhibition	ULK1	Indirect	SH-SY5Y (neuroblastoma)	(63)
miR-595						

### Cancer Cell Survival and Growth

As discussed above, autophagy competence is important for the growth and survival of cancer cells. A number of miRNAs were shown to regulate autophagy and control tumor cell growth and proliferation.

Expression of a number of miRNAs with autophagy-related targets resulted in growth inhibition in different cancer cell types: For example, overexpression of miR-143 inhibited proliferation of H1299 non-small lung cancer cells, and ATG2b was identified as an autophagy-related direct target of the miRNA (209). Overexpression of miR-9*-*3p in medullary thyroid cancer cell lines (TT and MZ-CRC-1 cells) decreased cellular levels of several autophagy-related proteins, including ATG5, PIK3C3, mTOR, and LAMP1, and inhibited autophagy, leading to G2 arrest and cell death (167). In another study, miR-502 inhibited autophagy through RAB1B and p53 targeting, and its overexpression suppressed colon cancer cell cycle progression and cell growth *in vitro* and in a tumor xenograft model (94). Von Hippel–Lindau (VHL) tumor suppressor is lost in the majority of renal cancers. A VHL-regulated miRNA, miR-204, blocked autophagy through miRNA-mediated downregulation of LC3B and suppressed growth of renal clear cell carcinoma (RCC) both in *in vitro* tests and *in vivo* in mice (220). VHL also repressed another protein involved in RCC growth, namely transient receptor potential melastatin 3 (TRPM3) through direct targeting by miR-204 (219). In fact, TRPM3 is a non-selective channel that is permeable to calcium and other cations. miR-204 directly targeted another TRPM3 regulator, CAV1, as well. On the other hand, overexpression of TRPM3 in RCC cells caused a Ca^2+^ influx that activated the calcium/calmodulin-dependent protein kinase kinase 2 (CAMKK2) and AMPK, which in turn activated ULK1 and triggered autophagy. TRPM3-mediated cation fluxes inhibited miR-214, an miRNA that directly targets LC3A and LC3B and inhibits autophagy. Therefore, an interplay between VHL and TRPM3 involving two miRNAs, namely miR-204 and miR-214, controls autophagy activation and renal cell carcinoma growth (219). Another miRNA-related autophagy control mechanism involves Yin Yang 1 (YY1), a transcription factor and an epigenetic regulator that is upregulated in various cancer types. miR-372, which was subject to epigenetic regulation by YY1, was found to target the autophagy receptor SQSTM1/p62 in a direct manner (229).

Under nutrient starvation condition, YY1 suppressed miR-372 expression, leading to SQSTM1/p62 expression and subsequent autophagy in breast cancer cell lines. Overexpression of miR-372 blocked autophagy activation and inhibited breast cancer xenograft growth *in vivo*, underlining the importance of YY1-mediated miR-372 suppression and autophagy for cancer cell proliferation (229). Other intricate connections also exist between autophagy-related miRNAs and cancer. Long non-coding RNA (lncRNA) PTENP1 is a pseudogene of the tumor-suppressor PTEN gene. Both PTENP1 and PTEN are downregulated in HCC cells. Interestingly, PTENP1 serves as a decoy for PTEN-targeting miRNAs, including miR-17, miR-19b, and miR-20a. These miRNAs also targeted PHLPP (a negative AKT regulator) and autophagy genes ULK1, ATG7, and p62. Overexpression of PTENP1 in HCC cells elevated the levels of PTENP1 and PTEN and suppressed growth-stimulating and autophagy-inhibiting PI3K/AKT pathway, as well as it suppressed cell proliferation and invasion and migration. Under these conditions, autophagy and apoptosis were induced. Mice experiments supported these findings: Vector-mediated introduction of PTENP1 into mice-mitigated HCC growth, attenuated cell proliferation, and triggered autophagy and apoptosis (171). Autophagy-mediated degradation of oncogenic or tumor suppressive molecules may also be manipulated by autophagy controlling miRNAs. One such example involves miR-125b1, an miRNA that is highly expressed in acute promyelocytic leukemia. miR-125b1 blocked proteolysis of the PML-RARA oncogenic protein by the autolysosomal system and contributed to the inhibition of leukemia differentiation (203). In this study, DNA damage-regulated autophagy modulator 2 (DRAM2), a critical regulator of autophagy, was described as a novel autophagy-related target of miR-125b1 (203). In another report, authors provided evidence that the oncomir miR-224 that promoted hepatoma cell migration and tumor formation was selectively recruited to autophagosomes, and the miRNA itself was degraded by autophagy (102). miR-224 affected tumor formation through silencing of Smad4. Importantly, impaired autophagy correlated with miR-224 accumulation and poor overall survival rate in HCC patients (102). Another recent study introduced miR-18A and an RNA-binding protein, hnRNP A1, as a target of autophagic degradation. Tumor-suppressor miR-18a is an apoptosis inducer in colon cancer cells, and this effect depended on the presence of hnRNP A1. The ribonucleoprotein was responsible for the stabilization of cyclin D1 and CTGF [or insulin-like growth factor-binding protein 8 (IGFBP-8)] mRNAs, and spared cancer cells from apoptosis. In order to limit tumor growth and promote cell death, miR-18a directly bound to hnRNP A1 and made it available for degradation by the autophagic machinery (173). Ge et al. reported that miR-100 overexpression resulted in death of HCC cells. Cell death depended on the activation of ATG7-dependent but *BECN1*-independent autophagy by the miRNA. For autophagy induction, miR-100 suppressed the expression of *mTOR and IGF-*1*R* by binding to their 3′-untranslated regions. Consistently, mice xenograft experiments revealed that miR-100 inhibited *in vivo* growth of HCC cells. Moreover, a correlation between miR-100 downregulation and upregulation of the autophagy receptor and target SQSTM1/p62 protein was observed in human HCC tissue samples compared to controls (196). Conversely, downregulation of miR-10a activated autophagy, apoptosis, and cell death in glioma cells (168). While expressed in low levels in the normal brain, the miRNA was found to be upregulated in glioma tissues and cells. miRNA upregulation correlated with poor prognosis. Inhibition of the miRNA led to cell cycle arrest, senescence, autophagy, apoptosis cell death, and reduced glioma growth in a mouse model *in vivo* (168). BCL-2L11/Bim, TFAP2C/AP-2γ, CDKN1A/p21, and CDKN2A/p16 were identified as relevant and direct targets of the miRNA in this context. Moreover, especially in glioma cells that were apoptosis-defective but still dying upon miRNA inhibition, strong autophagy activation was observed.

Since CDKN2A/p16 downregulation should lead to the suppression of the alternative reading frame products of the same gene, namely p14/p19ARF and mitochondrial smARF, authors suggested that these proteins might be instrumental in autophagic cell death activation following miRNA inhibition (234). Indeed, loss of the p16 and ARF-encoding CDKN2A gene was observed in around half of all gliomas, possibly contributing to autophagic cell death aversion during growth of the tumor (234).

### Cancer Cell Metabolism

Several studies in the literature implicated autophagy-related miRNAs in the regulation of metabolism and metabolic stress responses of cancer cells.

For example, *MIR290–295* cluster members (miR-291*-*3p, miR-291*-*5p, miR-292*-*3p, miR-292*-*5p, miR-294, and miR-295) targeted ATG7 and ULK1 on their 3′-UTR sequences, and reduced their protein levels in melanoma cells (227). Glucose starvation-induced cell death of metastatic B16F1 melanoma cells depended on their autophagic activity, and autophagy inhibition by miRNAs conferred resistance to death. Therefore, resistance to metabolic stress-induced death by *MIR290–295* cluster was a result of autophagy inhibition by these miRNAs (227). Another miRNA that had an impact on cellular metabolism was miR-124. This miRNA was mainly downregulated in colorectal adenoma and cancer specimen. miR-124 targeted polypyrimidine tract-binding protein 1 (PTB1), a protein that controls splicing of pyruvate kinase muscles to isoform 1 or isoform 2 (*PKM1* and *PKM*2) (201). PKM1 is mostly expressed in normal cells and tissues, where it stimulates oxidative phosphorylation. On the other hand, PKM2 is largely expressed in proliferating cells, including cancer cells, and it promotes glycolysis even under oxygen-rich conditions, supporting cancer cell metabolism and growth. Through suppression of PTB1, miR-124 induced a switch between PKM isoforms, from isoform PKM2 to PKM1, and increased oxidative phosphorylation and reactive oxygen accumulation in cancer cells. Consequently, ectopic expression of the miRNA or knockdown of PTB1 induced autophagy and apoptosis in colon cancer cells in *in vitro* and mice (201). miR-126 was downregulated in malignant mesothelioma tissues, and its expression was shown suppress tumor growth, possibly due to its effects on cancer cell metabolism. miR-126 suppressed IRS1, decreased glucose uptake, and caused energy deprivation that in turn switched on AMPK, leading to the activation of ULK1 (204). Moreover, miR-126 affected levels of other metabolism-related proteins, such as pyruvate dehydrogenase kinase and acetyl-CoA-citrate. These signals and metabolic changes that were triggered by the miRNA led to autophagy activation and inhibition of cancer growth both in *in vitro* cell culture and *in vivo* tests (204). Expression of another metabolism-related miRNA, miR-144 was found lower in lung cancer cell lines A549 and H460. Overexpression of the miRNA in these tumor cells was sufficient to block their proliferation and to promote autophagy and apoptosis (210). The authors identified TIGAR, a p53-induced regulator of glycolysis and apoptosis, as a direct target of the miRNA. TIGAR was shown to be important for rewiring of tumor cell energy metabolism and reduction of oxidative burden in cancer cells. Indeed, knockdown of TIGAR phenocopied the effects of the miRNA on cell growth, autophagy, and apoptosis. These results suggest that downregulation of miR-144 might be the result of a positive selection for TIGAR expression in lung cancer cells (210).

### Hypoxia Responses

Tumor cells face hypoxia as a result of abnormal vascularization and irregular blood supply. Under these circumstances, hypoxic tumor cells rely on autophagy for survival. A number of miRNAs were reported to control hypoxia-induced responses, including those that regulated autophagy in this context.

Upon hypoxia treatment, miR-124 and miR-144 were downregulated in DU145 and PC3 prostate cancer cell lines (202). Overexpression of these miRNAs reduced hypoxia-induced autophagy and enhanced radiation-induced cell death in prostate cancer cells (202). Authors claimed that suppression of the oncogene PIM1 was important for the observed effects. Another miRNA that was induced by hypoxia was miR-96. Expression of miR-96 in prostate cancer cells to moderate levels induced autophagy through direct suppression of mTOR. Yet, higher levels of the miRNA could also block ATG7 expression, therefore to explain these observations, authors proposed a miRNA level-dependent autophagy regulation model that prevented of autophagy hyperactivation during hypoxia. Indeed, in a series of prostate cancer tissues, miR-96 expression inversely correlated with mTOR and ATG7 (85). In Huh7 and Hep3B HCC cell lines, miR-375 expression was decreased following hypoxia treatment, and miR-375 levels were lower in HCC specimen compared to normal liver tissues (81). Interestingly, miR-375 suppressed prosurvival autophagy under hypoxia condition through targeting of ATG7 3′-UTR.

As stated above, autophagy is the main cellular clearance mechanism that eliminates damaged mitochondria in cells. As a consequence, overexpression of the miRNA blocked mitochondrial autophagy and sensitized HCC cells to hypoxia-induced mitochondrial cell death. Likewise, hypoxia led to the downregulation of miR-224*-*3p in glioma tissues. In cellular systems, expression of miR-224*-*3p abolished hypoxia-induced autophagy, whereas knocking down endogenous miR-224*-*3p increased autophagic activity under normoxia (77). miR-224*-*3p was shown to block autophagy by directly suppressing ATG5 and FIP200. Furthermore, the study showed that upregulation of the miRNA potentiated hypoxia-related cell death *in vitro* and inhibited glioblastoma tumor growth *in vivo*. In support of this observation, miR-224*-*3p levels inversely correlated with ATG5 and FIP200 expression in human glioma tissues (77). Another outcome of hypoxia in glioma cells was the stimulation of IL6 production and cytokine-mediated autophagy activation (213). In line with this, the amount of IL6 correlated with HIF1A levels and tumor grade in glioma tissues. In glioma cellular models, IL6-STAT3 axis led to the upregulation of an miRNA, miR-155*-*3p, and stimulated autophagy through a rather indirect manner. miR-155*-*3p directly targeted and decreased the levels of CREB3 inhibitor protein, CREBRF. Downregulation of CREBRF resulted in a CREB3-dependent increase in ATG5 transcription and autophagy stimulation was the end result. The role of the signaling pathway involving miR-155*-*3p in glioma cell survival was confirmed *in vitro* in cells, as well as *in vivo* in a tumor xenograft model. Blocking of IL6, hence autophagy inhibition, by antibody drugs alone or in combination with temozolomide (a first-line drug for glioma treatment) decreased cancer cell survival and the tumor burden. In contrast with miR-155*-*3p, complementary strand of the mature miR-155, namely miR-155*-*5p, was reported to block autophagy through downregulation of mTOR pathway components RHEB, RICTOR, and RPS6KB2, conducting cells to cycle arrest (212). Therefore, control of stability of either miR-155*-*3p *or -*5p strands of the same miRNA duplex might determine the final autophagy-related outcome under hypoxia. Alternatively, the competition between the two strands might determine whether autophagy will be inhibited or activated under hypoxia stress.

### Angiogenesis

Considering the importance of the autophagic activity for endothelial cell function and angiogenesis, one of the roles of hypoxia-induced autophagy in the cancer context is related to tumor neovascularization. Obviously, some of the autophagy-regulating miRNAs were shown to control the contribution of autophagy on the survival, growth, and spread of endothelial cells, having a direct impact on tumor vascularization.

For example, inhibition of an miRNA, miR-195, that is capable of targeting the autophagy protein GABARAPL1, stimulated autophagy in endothelial progenitor cells, promoted cell proliferation, migration, and angiogenesis under hypoxia (235). Addition of 3-MA was able to block all these cellular outcomes, pointing out to their autophagy dependence (235). miR-212, an miRNA that was downregulated in prostate cancer, inhibited autophagy through its direct effects on autophagy activator SIRT1 (222). Under these conditions, angiogenesis was suppressed and cancer cells were driven to senescence (222). On the other hand, inhibition of miR-130a correlated with autophagy induction through an RUNX3-*BECN1*/Beclin 1 axis and potentiated death of endothelial progenitor cells (236). High or fluctuating glucose levels had a similar effect on endothelial cells. Glucose level fluctuation led to an increase in miR-1273g*-*3p levels, which then, induced endothelial cell autophagy and blocked proliferation and migration of cells (237).

Above-mentioned studies give hints about the role of miRNA-autophagy connections in the regulation of endothelial cell homeostasis and angiogenesis *in vitro*. Further controlled *in vivo* studies are required to strengthen the link and establish their relevance to tumor vascularization.

### Cancer Cell Migration and Metastasis

Connections that exist between autophagy pathways and cellular migration also affect cell motility, invasion, and metastatic spread of cancer cells. Some of the miRNAs that regulate autophagy also had an influence on cancer cell migration and metastasis. Unfortunately, in most of these studies, a direct role for autophagy on migration was not established, yet there are hints in the current literature about an autophagy connection.

In some studies, miRNAs that attenuated migration and metastasis also targeted autophagy. For example, in colorectal cancer tissues, miR-140*-*5p levels inversely correlated with tumor progression toward invasion and metastasis. miR-140*-*5p directly targeted Smad2 that is involved in cancer stem cell maintenance and EMT and the autophagy protein ATG12 (207). The end result was suppression of autophagy, blockage of colon cancer cell proliferation and invasion *in vitro*, and inhibition of tumor formation and metastasis *in vivo* (207). In osteosarcoma cells, miR-22 downregulated cisplatin and doxorubicin-induced autophagy, and HMGB1 was identified as an autophagy-related target of the miRNA (181). Under these conditions, miRNA overexpression inhibited cellular proliferation, colony formation, *in vitro* migration, and transwell invasion capacity of cancer cells (181). These studies suggest that inhibition of autophagic activity by miRNAs may contribute to their anti-metastatic effects.

On the other hand, in some other contexts, prevention of migration and metastasis correlated with autophagy activation. For instance, miR-638, an miRNA that is an overexpressed miRNA in metastatic melanomas, increased proliferation and colony formation capacity of melanoma cells (233). Additionally, miR-638 overexpressing cells performed better in *in vitro* migration and invasion tests and in *in vivo* metastasis experiments. Aggressive behavior of melanoma cells depended on the suppressive effects of miR-638 on its target gene TP53INP2, a TP53-inducible nuclear protein that serves as a scaffold for autophagosome formation (233, 238). Antagomir-mediated neutralization of the miRNA led to the upregulation of its target genes and triggered p53-dependent autophagy and apoptosis (233) Therefore, miR-638 protected melanoma cells from autophagy and apoptosis to promote invasion and metastasis. While investigating the factors regulating ovarian cancer cell migration, Ferraresi et al. discovered that several miRNAs that were deregulated in response to IL6 and resveratrol (a polyphenolic compound inducer of autophagy) treatments (239). Six miRNAs that were regulated in an opposite manner by IL6 and resveratrol, namely miR-1305, miR-1260a, miR-141-3p, miR-424-5p, miR-15a-5p, and miR-7-5p, had as a common target, ARH-I (DIRAS3).

The protein encoded by this gene is a Ras homolog GTPase and a tumor suppressor in ovarian cancer, and it was shown to inhibit cell migration and stimulate autophagy and dormancy in this cancer type through its interaction with Beclin 1 (240). In this setting, IL6 treatment prevented LC3-positive vacuole accumulation and promoted cellular motility, while resveratrol had the opposite effect on both autophagy and cell migration (239). In some contexts, autophagy was shown to be responsible for direct elimination of miRNAs that promoted migration (102). These results point out to an anti-metastatic role of autophagy under certain circumstances.

### miRNAs As Cancer Biomarkers

Among the miRNAs that are involved in autophagy regulation, some of them were introduced as potential tumor biomarkers. For example, miR-221*/*222 was evaluated as a prognosis predictive biomarker in the plasma of patients with breast cancer that have been treated with a neoadjuvant chemotherapy (241). On the other hand, miR-205 and miR-342 levels were found to be significantly low in triple-negative breast cancer tissues (242). Again in triple-negative breast cancers, miR-155, miR-493, miR-30e, and miR-27a were tested as prognostic biomarkers, and upregulation of miR-155 and miR-493 was associated with a better patient outcome, while suppression of miR-30e and miR-27a correlated with a worse outcome (243). In ovarian cancers, a decrease in miR-152 levels was associated with cisplatin resistance (211) and miR-29b expression correlated with better prognosis (244). On the other hand, in prostate tumors, a decrease in miR-212 expression in tumor tissues and sera of patients indicated a diagnostic potential for this miRNA (222). There are several other studies implicating autophagy-related miRNAs in cancer diagnosis and in some cases reporting their prognosis prediction potential. See Table 2 for some examples of autophagy-related miRNAs with biomarker potential.

**Table 2:** **Autophagy-related microRNAs (miRNAs) as biomarkers**.

miRNAs	miRNA status in cancer	Prognostic or diagnostic marker	Tissue	Reference
miR-16	Decreased	Prognosis	Melanoma	(245)
miR-16	Decreased	Prognosis	Childhood ALL	(246)
miR-17*-*5p	Increased	Diagnosis	Gastric cancer	(247)
miR-17*-*5p	Increased	Diagnosis	Nasopharyngeal cancer	(248)
miR-21	Increased	Diagnosis	Diffuse large B cell lymphoma	(249)
miR-26b	Decreased	Prognosis	Cervical cancer	(250)
miR-29b	Decreased	Prognosis	Ovarian cancer	(244)
miR-30d	Increased	Diagnosis	Low-grade serous ovarian cancer	(251)
miR-34a	Decreased	Diagnosis	Diffuse large B cell lymphoma	(252)
miR-140*-*5p	Decreased	Prognosis	Colorectal cancer	(207)
miR-143	Decreased	Diagnosis	Pancreas cancer	(253)
miR-155	Increased	Diagnosis	Diffuse large B cell lymphoma	(252)
miR-155	Increased	Diagnosis	Diffuse large B cell lymphoma	(249)
miR-155	Decreased	Diagnosis	Pancreas cancer	(253)
miR-183, miR-375	Increased	Prognosis	Sporadic medullary thyroid cancer	(254)
miR-205	Decreased	Diagnosis	Triple negative breast cancer	(242)
miR-210	Increased	Prognosis	Melanoma	(255)
miR-210	Increased	Diagnosis	Diffuse large B cell lymphoma	(249)
miR-212	Decreased	Diagnosis	Prostate cancer	(222)
miR-216a	Decreased	Diagnosis	Pancreas cancer	(253)
miR-221*/*222	Increased	Diagnosis	HR-negative breast cancer	(241)
miR-224*-*3p	Increased	Diagnosis	HPV-positive cervical cancer	(226)
miR-340	Decreased	Prognosis	Glioblastoma	(228)
miR-409*-*3p	Decreased	Prognosis	Gastric cancer	(256)

Although the contribution of autophagy competence and activity was not studied in all biomarker studies, it is possible that autophagy-related effects of the miRNAs might be contributing to the tumor behavior and disease prognosis. Correlative analyses that combine molecular and cellular data on autophagy are required to establish and confirm the relationship between autophagic capacity of tumors and diagnostic/prognostic value of autophagy-related miRNAs.

### Autophagy-Related miRNAs and Response to Cancer Treatment

#### Response to Radiotherapy

Radiation treatment is one of the standard treatment modalities for many cancer types. Radiotherapy involves the use of ionizing radiation at doses that damage cancer cells. Since normal cells in the surrounding tissues may also be affected, dose adjustments and focused applications are important issues to be considered to obtain effective treatment protocols with minimal side effects. Mechanism of action of radiation in cancer cells include generation of oxygen radicals, damage to organelles such as mitochondria and ER, and direct and oxidative damage to DNA and other cellular components (257) All these insults trigger autophagy responses as well. Indeed, autophagy emerges as one of the factors that can influence the efficacy of radiation treatment of cancer (257, 258). Evidently, autophagy-regulating miRNAs have the capacity to modify responses of cancer cells to radiation treatment.

For example, miR-23b was shown to target ATG12 and inhibit autophagy, and overexpression of the miRNA sensitized pancreas cancer cells to radiation (184). In another study, miR-216a downregulation correlated with autophagy activation in radiation-resistant prostate cancer cells through depression of *BECN1*/Beclin 1, and forced expression of the miRNA led to radiosensitivity and cell death (73).

On the other hand, miR-32 was shown to induce autophagy through suppression of autophagy inhibitor DAB2IP, enhancing prostate cancer cell survival following radiation treatment (192). Strikingly in some contexts and tumor types, autophagy seems to confer resistance to radiation-induced cancer cell death. For instance, inhibition of miR-17 that targeted ATG7 activated autophagy and sensitized U373-MG glioma cells to low-dose ionizing radiation treatment, affecting their long-term viability (83). In another study, miR-199a*-*5p increased basal and radiation-induced autophagy breast cancer cells, and autophagy activation by this miRNA in MCF-7 cells correlated with sensitivity to radiation (70).

The effects of autophagy-related miRNAs on radiation responses were summarized in Table 3. Whether discrepancies between these observations are a result of a switch between protective and prosurvival autophagy and its autophagic, apoptotic, or necrotic cell death promoting role is not clear to date and further molecular studies are required. Nevertheless, altogether these studies underline the fact that autophagy manipulation in a context-dependent manner might potentiate responses of cancer cells to radiotherapy and improve treatment outcomes.

**Table 3:** **Effect of autophagy-related microRNAs (miRNAs) on radiotherapy**.

miRNAs	miRNA status in cancer	Effect on autophagy	Autophagy-related target	Effect on radiotherapy	Tested cell line (tissue origin)	Reference
miR-17	Upregulated	Inhibition	ATG7	Radioresistance	U373 (glioblastoma)	(83)
miR-21	Upregulated	Inhibition	N.D.	Radioresistance	U373, U87 (glioblastoma)	(175)
miR-21	Upregulated	Inhibition	PTEN	Radioresistance	HeLa, siHa (cervical)	(177)
miR-23b	Downregulated	Inhibition	ATG12	Radiosensitivity	BxPC3 (pancreas)	(184)
miR-30b	Downregulated	Activation	BECN1	N.D.	SH-SY5Y (neuroblastoma)	(190)
miR-32	N.D.	Inhibition	DAB2IP	Radioresistance	PC3, DU145 (prostate)	(192)
miR-101	Upregulated	Inhibition	STMN1	Radiosensitivity	CNE-2, 5–8 F (nasopharyngeal carcinoma)	(199)
miR-199a*-*5p	N.D.	Inhibition	BECN1, DRAM1	Radiosensitivity	MDA-MB-231 (breast)	(70)
miR-200c	N.D.	Activation	UBQLN1	Radiosensitivity	MDA-MB-231 (breast)	(218)
miR-205	N.D.	Inhibition	TP53INP1	Radiosensitivity	DU145, LNCaP (prostate)	(221)
miR-216a	Upregulated	Inhibition	BECN1	Radiosensitivity	PANC-1 (pancreas)	(80)

#### Response to Chemotherapy

Most of the tested chemotherapy agents have been shown to induce autophagy in cancer cells, and miRNAs that control autophagic activity were reported to affect susceptibility of cancer cells to cancer drugs.

For example, miR-101 and miR-199a*-*5p that both had inhibitory effects on autophagy potentiated liver cancer cell death by cisplatin (87, 197). Similarly, in liver cancer cells, toxic effects of another chemotherapy agent, doxorubicin, were increased when miR-101 was overexpressed (198). In lung cancer cells, miR-24*-*3p increased sensitivity to etoposide and cisplatin, miR-200b to docetaxel, miR-216B to paclitaxel, and miR-487*-*5p to temozolomide (97, 217, 224). All of the above miRNAs were shown to block autophagy in the lung cancer context. On the other hand, miR-17 and miR-16 increased paclitaxel sensitivity of lung cancer cells through simultaneous downregulation of autophagy and activation of apoptosis following *BECN1*/Beclin 1 and BCL-2 suppression, respectively (170). Similar combined effects of miRNAs and chemotherapy agents were observed in medullablastomas, multiple myelomas, chronic myeloid leukemias, gliomas, cervix, ovary, breast, prostate and head and neck cancers, esophageal, gastric, colorectal, and thyroid carcinomas. Importantly, same miRNAs were able to show chemotherapy potentiation effects in more than one cancer type in independent studies. For example, miR-21 augmented chemotherapy responses of various cancer drugs on liver, breast, and head and neck cancers, indicating that observed effects may well be independent of cancer type (178–180, 183). Table 4 summarizes the effects of autophagy-regulating miRNAs on chemotherapy responses.

**Table 4:** **Effect of autophagy-related microRNAs (miRNAs) on chemotherapy**.

miRNAs	miRNA status in cancer	Effect on autophagy	Autophagy-related target	miRNA effect on chemotherapy	Chemotherapeutic agent	Tested cell line (tissue origin)	Reference
miR-let7f1	N.D.	Inhibition	HMGB1	Chemosensitivity	Cisplatin	D425, UW228 (medullablastoma)	(165)
miR-15a*/*16	N.D.	Activation	RICTOR	Chemosensitivity	Camptothecin	HeLa (cervical cancer)	(169)
miR-16 miR-17	Downregulated	Activation	BCL-2	Chemoresistance	Paclitaxel	A549-T24 (lung cancer)	(170)
miR-17	Upregulated	Inhibition	ATG7	Chemosensitivity	Temozolomide	U373 (glioma)	(83)
miR-17*-*5p	Downregulated	Inhibition	BECN1	Chemosensitivity	Paclitaxel	A549-T24 (lung cancer)	(172)
miR-21	Upregulated	Activation	PTEN	Chemosensitivity	Sorafenib	Huh7, HepG2 (liver cancer)	(178)
miR-21	N.D.	Inhibition	PTEN	Chemoresistance	Tamoxifen Fulvestrant	MCF-7 (breast cancer)	(179)
miR-21	Upregulated	N.D.	PDCD4	Chemosensitivity	Cisplatin	Tca8113 (tongue squamous cell carcinoma)	(180)
miR-21	Downregulated	N.D.	N.D.	Chemosensitivity	Cisplatin	Tca8113 (tongue squamous cell carcinoma)	(183)
miR-22	N.D.	Inhibition	BTG1	Chemosensitivity	5-FU	SW620, RKO (colorectal cancer)	(182)
miR-23a	Upregulated	N.D.	TOP2B	Chemoresistance	Cisplatin	Tca8113 (tongue squamous cell carcinoma)	(183)
miR-23b*-*3p	Downregulated	Inhibition	ATG12, HMGB2	Chemosensitivity	5-FU, Cisplatin	SGC7901/VCR (gastric cancer)	(185)
miR-24*-*3p	Downregulated	Inhibition	ATG4A	Chemosensitivity	Etoposide Cisplatin	H446/EP (lung cancer)	(80)
miR-25	N.D.	Inhibition	ULK1	Chemosensitivity	Isoliquiritigenin	MCF-7 (breast cancer)	(61)
miR-29b	Downregulated	Inhibition	PSME4	Chemosensitivity	Bortezomib	AMCL1, AMCL2 (multiple myeloma)	(187)
miR-30b	N.D.	Inhibition	BECN1	Chemosensitivity	Imatinib	K562 (CML)	(65)
miR-30a	Downregulated	Inhibition	BECN1	Chemosensitivity	Cisplatin	HeLa (cervical cancer)	(188)
miR-30d	N.D.	Inhibition	BECN1	Chemosensitivity	Cisplatin	SW1736, 8305 C (anaplastic tyroid carcinoma)	(191)
miR-30a	N.D.	Inhibition	BECN1	Chemosensitivity	Sorafenib	786-0, A489 (renal carcinoma)	(189)
miR-30a	Downregulated	Inhibition	BECN1	Chemosensitivity	Doxorubicin	MG-63 (osteosarcoma)	(66)
miR-101	N.D.	Inhibition	STMN1 RAB5A Atg4D mammalian TOR (mTOR)	Chemosensitivity	Cisplatin	HepG2 (liver cancer)	(197)
miR-101	N.D.	Inhibition	STMN1 RAB5A Atg4D	Chemosensitivity	Etoposide	MCF-7 (breast cancer)	(89)
miR-101	Downregulated	Inhibition	EZH2	Chemosensitivity	Doxorubicin	HepG2 (liver cancer)	(198)
miR-138	N.D.	Activation	BIM	Chemosensitivity	Temozolomide	LN-308, ZH-305 (glioblastoma)	(206)
miR-143	Downregulated	Inhibition	GABARAPL1	Chemosensitivity	Qercetin	AGS, MKN28 (gastric cancer)	(208)
miR-143	Downregulated	Inhibition	ATG2B	Chemosensitivity	Doxorubicin	SAOS-2-Dox, U2OS-Dox (osteosarcoma)	(259)
miR-152	Downregulated	Inhibition	ATG14	Chemosensitivity	Cisplatin Doxorubicin	A2780/CP70, SKOV3/DDP (ovarian cancer)	(211)
miR-181a	N.D.	Inhibition	ATG5	Chemosensitivity	Cisplatin	MCF-7 (breast cancer)	(75)
miR-181a	N.D.	Inhibition	ATG5	Chemosensitivity	Cisplatin	SGC7901/CDDP (gastric cancer)	(214)
miR-193b	Upregulated	Activation	STMN1	Chemosensitivity	5-FU	KYSE450 (esophageal cancer)	(216)
miR-199a*-*5p	Downregulated	Inhibition	ATG7	Chemosensitivity	Cisplatin	Huh7, HepG2 (liver cancer)	(87)
miR-205	N.D.	Inhibition	RAB27A, LAMP3	Chemosensitivity	Cisplatin	DU145 (prostate cancer)	(95)
miR-200b	N.D.	Inhibition	ATG12	Chemosensitivity	Docetaxel	SPC-A1/DTX, H1299/DTX (lung cancer)	(217)
miR-214	Downregulated	Inhibition	UCP2	Chemosensitivity	Tamoxifen Fulvestrant	MCF-7/LCC9 (breast cancer)	(223)
miR-214	Upregulated	N.D.	N.D.	Chemoresistance	Cisplatin	Tca8113 (tongue squamous cell carcinoma)	(183)
miR-216b	Downregulated	Inhibition	BECN1	Chemosensitivity	Paclitaxel	A549, Calu-3 (lung cancer)	(224)
miR-218	Downregulated	Inhibition	HMGB1	Chemosensitivity	Paclitaxel	RL95-2 (endometrial carcinoma)	(225)
miR-409*-*3p	Downregulated	Inhibition	BECN1	Chemosensitivity	Oxaliplatin	Lovo Oxa R (colorectal cancer)	(72)
miR-451a	N.D.	Inhibition	N.D.	Chemosensitivity	Tamoxifen	MCF-7/LCC2 (breast cancer)	(231)
miR-487*-*5p	Upregulated	Inhibition	LAMP2	Chemoresistance	Temozolomide	A549, H1299 (lung cancer)	(97)

## Conclusion

Studies that are published so far about the regulation of autophagy by miRNAs start to reveal a general picture about this emerging field. In human cells, there are around 2,000 miRNAs and 25,000 protein-coding genes. Approximately 60% of all these protein-coding genes are predicted to be controlled by miRNAs (16). Yet, accumulating data in the literature indicate that genes of almost all proteins that are involved in autophagosome formation and maturation as well as components of autophagy-related signaling pathways (e.g., AMPK, AKT, and mTOR pathways) are strictly controlled by several miRNAs (Table 1). Some autophagy genes may even be targeted by more than one miRNA that have divergent responses to stress stimuli [e.g., Ref. (64, 67)].

Since autophagy is an evolutionarily well-preserved pathway in all organisms from yeast to man, and it is essential for cellular and organismal homeostasis and survival, strict control of autophagy at every level should be an expected outcome.

Upregulation or downregulation of miRNAs was observed in almost all types of cancer, indicating that proper functioning of miRNA networks ensure normal growth and behavior of cells. A single miRNA is able to control dozens of genes, changing thresholds and responsivity or even function of signaling pathways and signal-related events. Modulation of cellular stress and death responses and regulation of cell growth, cell-extracellular matrix, and cell-to-cell interactions as well as cellular migration capacities are all subject to control by miRNAs. Therefore, deregulation of miRNA networks might directly contribute to cancer cell formation, EMT, neovascularization, tissue invasion, and metastasis. Even some miRNAs were classified as oncogenes (oncomirs) and others as tumor suppressors. A number of miRNAs that were shown to play a role in cancer biology were also involved in autophagy regulation, and many of them were reported to directly target autophagy-related genes.

Autophagy abnormalities are generally either the cause or an exacerbating factor in a large majority of diseases in man and in other organisms. And cancer is no exception. Autophagy plays a role in various steps of cancer formation, growth, and spread. Not surprisingly, there is a growing literature about deregulation of autophagy-related miRNAs in cancer. Direct contribution of autophagy abnormalities to phenotypes that were observed following miRNA deregulations was not established in some cancer-related publications, and it is possible that contribution of autophagy is somewhat indirect in some of these studies. Nevertheless, it is unimaginable that autophagy-related effects of these miRNAs will be of no consequence to cancer cell behavior. Moreover, there is a rapidly growing literature about autophagy-related miRNA levels and sensitivity to chemotherapy or radiation treatment. In line with the prosurvival role of autophagy, in most of these studies, autophagy suppression by miRNAs was shown to sensitize cancer cells to therapy. In contrast, in some cases, aberrant activation of autophagy that correlated with changes in miRNA levels was itself detrimental for cancer cells and led to apoptotic, autophagic, or necrotic cell death. Therefore, miRNA manipulations through using mimics or antagomirs, or other strategies, might potentially be used as adjuvant therapies for cancer treatment. Advances in gene therapy protocols and improvement of gene delivery vehicles (e.g., new generation gene therapy viruses, liposomes, nanoparticles, etc.) might allow the use of miRNA manipulation strategies in cancer gene therapy trials.

Another important and more immediate use of miRNAs in oncology involves exploitation of their disease marker potential. In addition to allowing early and accurate diagnosis of cancer, miRNAs may be used to follow patient responses to therapy and relapses. In addition to tumor biopsy materials, miRNAs can potentially be detected in any bodily fluid, including blood, urine, saliva, etc. Autophagy-related miRNAs were also found to be up- or downregulated in many cancers, and several studies point out to their potential use as biomarkers (Table 2).

In conclusion, autophagy-related miRNAs constitute a very important control layer on top of all other autophagy-regulatory mechanisms that were described so far. In the last few years, there is an exponential increase in the number of articles studying miRNA-autophagy connection. These efforts will eventually result in the construction of a detailed and functional map of autophagy-related miRNA networks. Accumulation of knowledge on miRNA-mediated control of autophagy under physiological and pathological conditions might lead to the development of new approaches that can be used for the diagnosis, treatment, and follow-up of serious health problems involving autophagy abnormalities, including cancer.

## Author Contributions

DG designed the structure of the review, wrote, and edited the review article. YA and DO wrote the review article prepared the tables. MK contributed to the preparation of the tables and prepared the figures.

## Conflict of Interest Statement

The authors declare that the research was conducted in the absence of any commercial or financial relationships that could be construed as a potential conflict of interest.
